# Navigating Mental Health Care: Latine Perceptions, Barriers, and Pathways to Engagement

**DOI:** 10.21203/rs.3.rs-7723932/v1

**Published:** 2025-12-22

**Authors:** Cristina Calderon, Lisa Ochoa-Frongia, Elysia Teruya, Rolando Del Pozo, Giselle Aguayo Ramirez, Maria E. Garcia

**Affiliations:** University of California, San Francisco; University of California, San Francisco; University of San Francisco; University of California, Davis; University of California, San Francisco; University of California, San Francisco

**Keywords:** Latine, Depression, Depression Diagnosis, Trust, Depression care engagement

## Abstract

**Background:**

Latine individuals in the U.S. experience similar or higher rates of depression compared to non-Hispanic Whites but remain less likely to receive timely or effective mental health care. While structural and cultural barriers have been well-documented, limited research has examined how Latine primary care patients conceptualize depression and depression care. This study explores the factors shaping engagement in depression care among Latine patients.

**Methods:**

We conducted a qualitative study of 10 Latine patients (13 interviews). Eligible participants included those who: (1) were ≥ 18 years old, (2) had received care in a primary care clinic between November 1, 2019 and March 31, 2020, (3) self-identified as Latine and spoke English or Spanish, and (4) had depressive symptoms (defined as a PHQ-2 score ≥3) and/or PHQ-9 (score ≥10) or had a diagnosis of depression on their problem list or an ICD-10 code for depression (F32.0 - F33.3) between November 1, 2019 to December 31, 2020. Semi-structured interviews were conducted in English or Spanish and analyzed using consensual qualitative research methods.

**Results:**

In addition to structural barriers like limited language concordant care, four themes emerged from our data; 1) importance of trust in primary care physicians (PCPs) and creating a safe space for discussing mental health; (2) reliance on culturally informed coping strategies to manage depressive symptoms; (3) variable understanding of depression diagnosis impacts care; and (4) beliefs about the etiology of depression may influence willingness to engage in care.

**Conclusions:**

Our findings show that trust, cultural values, and illness beliefs shape how Latine patients engage in depression care. Interventions that validate patient perspectives, address language barriers, and build longitudinal trust may promote more equitable, culturally responsive depression care. These findings offer guidance for health systems and PCPs aiming to improve depression care engagement and outcomes among Latine populations.

## Background

In the United States, Latine individuals experience similar[1] or higher[2] rates of depression to non-Hispanic Whites but are much less likely to receive depression care.[3] Prior studies have identified structural and cultural barriers to mental health care access among Latine individuals, including limited insurance coverage, cost, transportation challenges, lack of language-concordant care, preferences for social support networks, and stigma.[4][5][6] Furthermore, research has shown that Latine individuals with a psychiatric disorder are more likely than Black and White patients to report little or no perceived need for treatment, contributing to lower engagement in mental health care.[7]

Despite growing national efforts to improve mental health equity, depression care gaps for Latine populations persist, particularly within primary care settings, where most mental health concerns are first identified and managed.[8][9] While structural barriers such as insurance gaps and physician shortages are well-documented,[10] fewer studies have explored what happens once Latine patients are engaged in care. Specifically, there remains a need to understand how Latine patients experience communication around depression, how they interpret and respond to a diagnosis, and how they navigate treatment in the context of cultural and linguistic dynamics in primary care settings.[11]

These underexamined factors are critical; research demonstrates that culturally discordant care, limited language proficiency, and poor patient-physician communication can reduce engagement in treatment and exacerbate mental health inequities.[12][13][14] Moreover, disparities in care may persist beyond the initial point of access and inequities in patient-physician interactions may hinder care continuity and quality.[15][8] To address these gaps, this study explores how Latine primary care patients understand and experience depression-related care. Through qualitative interviews, we examine factors that may shape patients’ responses to their depression diagnosis and their decisions to engage, or not engage, in care.

## Methods

### Study Setting

Participants were recruited from an academic safety-net primary care clinic in San Francisco, California which serves approximately 8,000 adult patients. The patient population is predominantly female (55%), with the majority insured through Medi-Cal/Medicaid (61%), Medicare (19%), Healthy San Francisco (13%; an HMO health insurance program in San Francisco offered to providers of In-Home Support Services workers) and private insurance (1%). Nearly half of the patients identify as Latine (47%), others identify as Asian (22%), White (13%), Black/African American (11%), and Native Hawaiian/Pacific Islander (1%). Forty-eight percent of patients speak a primary language other than English, most commonly Spanish (36%) or Cantonese/Mandarin (10%).

### Purposive Participant Sampling and Recruitment

Eligible participants included those who: (1) were ≥ 18 years old, (2) had received care at the clinic between November 1, 2019 and March 31, 2020, (3) self-identified as Latine and spoke English or Spanish, and (4) had depressive symptoms (defined as a PHQ-2 score ≥ 3) and/or PHQ-9 (score ≥ 10) or had a diagnosis of depression on their problem list or an ICD-10 code for depression (F32.0 - F33.3) between November 1, 2019 to December 31, 2020. Eligible participants were identified through the electronic health record (EHR; Epic). The team contacted patients’ respective primary care physicians (PCP) for permission to contact eligible patients prior to recruitment via phone call.

The PHQ-2 and PHQ-9 are two validated and widely used measures of current depressive symptoms.[16][17] A PHQ-9 score ≥ 10 has high sensitivity (88%) and specificity (88%) for major depression.[18] A PHQ-9 score ≥ 10 is considered a positive depression screen and, if the diagnosis is confirmed, often triggers depression care and referral to integrated behavioral health services.

### Interviews

Study team members trained in qualitative research methods developed an interview guide informed by consensual qualitative research (CQR) theory,[19] the Capability, Opportunity, Motivation for Behavior change (COM-B) model,[20] and current literature on depression.[9] The interview guide (Appendix 1) featured questions about the patient’s socio-environmental context, emotional well-being, barriers and facilitators to mental health care, most recent encounters with their PCP, as well as opinions on general mental health and depression.

Two bilingual interviewers (R.D.P, C.C.) conducted semi-structured interviews in the participant’s preferred language (English or Spanish) which lasted approximately one hour. To understand how access to care may change over time, we offered participants the possibility of a second interview approximately a year later. All interviews were conducted virtually using Zoom, a video conferencing platform. Participants received a $25 gift card per interview, for a maximum total of $50. All audio-recordings were professionally transcribed and translated, if needed.

### Qualitative Analysis

We blended deductive and inductive thematic analysis methodologies to capture theoretically and clinically important concepts regarding barriers and facilitators to engaging in depression care. To develop the codebook, two coders began by individually coding the first transcript before collaborating on the identification of codes. The larger research team iteratively reviewed the codebook until a consensus was reached. Once we established the codebook (See Appendix 2), all transcripts were independently coded by 2–3 coders and reviewed by the group. The researchers applied consensual qualitative research theory to ensure coding was consistent amongst all members and used thematic analysis to identify emerging themes.[21]

We achieved thematic saturation[22] after analyzing data from the first five of the 13 transcripts, with subsequent interviews adding depth and richness to the data but no new themes. Data analysis and coding aggregation of all data were conducted using Dedoose, a qualitative analysis software.

This study received ethical approval from the University of California, San Francisco Institutional Review Board and informed consent to participate was obtained from all of the participants in the study.

## Results

Of 64 eligible individuals contacted, ten participated in the first interview in 2021 (8 Spanish-speakers, 2 English-speakers). In 2022, three individuals participated in an additional interview, for a total of 13 interviews (average length 52 minutes). Four participants were women and six were men. The median age was 45 years (range 31–60). Seven participants immigrated to the United States (three from Mexico, two from El Salvador and one each from Peru, and Guatemala). Five participants were currently employed. All ten participants had a baseline PHQ-9 score > = 10. The mean baseline PHQ-9 score was 15.1 (range 10–19).

In addition to structural barriers like limited language concordant care which have been well described in the literature, four themes emerged from our data; 1) importance of trust in primary care physicians (PCPs) and creating a safe space for discussing mental health; (2) reliance on culturally informed coping strategies to manage depressive symptoms; (3) variable understanding of depression diagnosis impacts care; and(4) beliefs about the etiology of depression may influence willingness to engage in care.

### Theme 1: Importance of trust in PCPs and creating a safe space for discussing mental health

Participants emphasized that trust in PCPs and a welcoming space were essential for discussing mental health concerns during primary care visits. They described various approaches PCPs used to foster trust, including taking a comprehensive approach to care and establishing personal connections.

Participants reported PCPs gained their trust by demonstrating attentiveness to both physical and emotional symptoms. One participant explained how his PCP’s thorough evaluations reassured him that he was receiving appropriate care, “I feel comfortable, he checks my heart, he checks my blood pressure, he checks my lungs– he checks my whole system and tells me that everything is fine, just a little bit of that and with that, I feel more at ease and I know what to do and what not to do” (I1 P6, Male). This created a sense of safety and openness that made it easier to bring up emotional concerns. Another participant described how small talk and probing questions contributed to trust-building, “She [my doctor] pays attention to my problems. Because whenever I go to this one, well, I go for another reason, but she always asks me something like ‘How are you today? What happened? Did you feel lonely?’” And all that. So, it has nothing to do with what I’m going for, but she always asks me that” (I1 P9, Female). These accounts underscore the value of small talk and inquiry in fostering trust during medical encounters, enabling patients to more openly discuss mental health symptoms.

Other participants described how trust developed through a strong personal connection with their PCP. One participant recalled feeling an immediate sense of rapport, despite cultural differences, “I felt like that connection…I saw that we had a lot in common in terms of thoughts. Even though we are not from the same country or anything like that…So I was able to open up a little, talk to her” (I1 P7, Male). This perspective highlights how shared viewpoints, regardless of cultural background, can contribute to trust. Another participant described how his PCP’s consistent support over time strengthened their relationship, “I’ve just had a good relationship with her. And I’ve already discussed what would be the most embarrassing possible scenarios with her and, you know, that’s how… I’m very comfortable with her… She’s proven to me that I can be comfortable with her, and it’s been that way for a while” (I1 P3, Male). For some, language concordance was essential in building trust. One participant explained how speaking with his provider in his preferred language enhanced their connection, “I feel good because -- ever since I met her and she was assigned to me, she has worked well with me…she speaks Spanish, she speaks English, she understands me” (I1 P2, Male).

### Theme 2: Reliance on culturally informed coping strategies to manage depressive symptoms

Participants described a range of strategies they use to manage depressive symptoms, often turning to complementary health practices, hobbies, and social support. They emphasized that when personal coping mechanisms were insufficient, they relied on their community and support networks for relief.

Many participants described using complementary care strategies to promote their mental health; these were often deeply rooted in their cultural backgrounds. One participant reflected on how his cultural connection to the ocean played a role in his mental health management, “I’m from Guerrero [Mexico], where you come and you go to see the sea and you have to throw everything in the water, let it be taken away to clean your head” (I2 P5, Male). Other participants preferred coping strategies that provided a distraction from their depressive symptoms. One participant explained how engaging in a hobby helped her shift focus, “I do something that, I don’t know, that I like…like painting things…I try to get my mind off things, to get out of it, not to think anymore” (I1 P8, Female). Similarly, another participant described using physical activity as a distraction, “get active, do something, I don’t know, clean the house…I try to do the most, the most -- distract my mind as much as I can” (I1 P6, Male).

In addition, participants emphasized the role of social connections to manage mental health symptoms. Some relied on their communities for direct support, while others sought companionship for distraction. One participant shared how his support network provided relief, “I know so many people in San Francisco, and I have such a strong support system in the DUI class, which I go to once a week every Wednesday. And if, you know, a hard day at work, I can go tell it to my counselor there or my educator there and just kind of blow off some real steam” (I1 P1, Male). Other participants emphasized how connecting with family or friends enhances mental health even if not directly addressing symptoms, “There are times that I call my brother, but I don’t tell him that I feel depressed, that I feel lonely or anything like that, I just call him and tell him it’s to talk, to see what’s going on” (I1 P7, Male). And “I try, as always, to look for friends, to talk to friends, I mean, but not about the problem, but as a way to, to distract myself from the problem.” (I1 P9, Female). These perspectives highlight the essential role of both formal and informal support systems in managing depressive symptoms.

### Theme 3: Variable understanding of depression diagnosis impacts care

Participant understanding of and reactions to their depression diagnosis varied. When asked about their diagnosis, responses ranged from clear recollection of discussions with their PCP to confusion or complete lack of awareness that depression had been diagnosed.

Some participants were not surprised to hear they had a depression diagnosis and recalled explicit discussions with their PCP. One participant described a thorough conversation with his PCP that included resources and professional referrals, “she [my PCP] discussed all that with me. She gave me forms, pamphlets, she had a psychiatrist come in and talk to me. And it went from there” (I1 P1, Male). Another participant agreed with her physician’s diagnosis and described how her PCP provided guidance on accessing therapy despite systemic barriers, “Yes, it was appropriate, because yes, sometimes I would get -- sometimes I would be depressed when I went to see my doctor and I would want to get therapy” (I1 P9, Female). For these participants, trust and engagement in the patient-provider relationship appeared to play a role in their understanding and acceptance of their diagnosis. These individuals also demonstrated a stronger understanding of the connection between their diagnosis, the discussions with their PCP, and the resources offered.

Other participants recalled conversations about their mental health symptoms but had not realized they had received a clinical diagnosis of depression. One participant recalled receiving support and referrals but noted that the explicit term “depression” had not been used, “Well, the doctor didn’t tell me exactly that I had depression…No, she listened to all my -- at that moment what I was going through, she also gave me a lot of advice, she put me in contact with places like that, [community-based organization that offers psychotherapy]. The thing is that she did tell me to go there. But she didn’t tell me at any moment that I was depressed” (I1 P8, Female). Another participant acknowledged that depression was listed in his medical record but did not recall a direct conversation confirming the diagnosis, “Well, the doctors did not tell me that I had depression, they told me that the depression I had was on the list, that it gave me a lot of pain, they gave me some medications, and with that I controlled it” (I1 P2, Male).

Similarly, other participants recalled less about a diagnosis, expressing confusion when asked. One participant questioned whether his diagnosis was based on a single difficult day or a routine screening tool rather than a formal diagnosis, “I don’t recall… I don’t remember the exact moment or visit that you’re speaking of. I’m a little bit confused about it myself, actually. Because you mentioned that at the beginning of the conversation. And I’m wondering was it just a bad day or probably a brief form that I sometimes fill out at the doctor? Maybe I just…News to me” (I1 P3, Male). Another participant was similarly confused, stating, “I didn’t really get the diagnosis…I went for -- I went to see the doctor, but she didn’t even see me [patient say another physician], (I1 P10, Female). These cases suggest a communication gap in which PCPs may discuss symptoms and document a diagnosis in medical records without ensuring patient understanding. It is unclear whether a depression diagnosis was not discussed or if the patient simply did not remember the discussion; regardless, these cases suggest a need for additional discussion to ensure patient understanding of their diagnosis to facilitate depression treatment engagement. Patient reported misunderstandings may reflect a broader issue of accessibility, rushed consultations, or a disconnect between the patient’s expectations and the PCP’s approach to discussing mental health conditions.

### Theme 4: Beliefs about the etiology of depression may influence willingness to engage in care.

Participants described two perceived etiologies of depression: as a result of a personal failing, and as a hereditary disease beyond their personal control. These perceptions appeared to shape participants’ willingness to engage with mental healthcare.

Some participants described depression as something within one’s control, often associating it with willpower and self-discipline. This belief was often associated with a low willingness to engage with prescribed mental health care. One participant framed depression as a product of the mind’s influence over the body, stating, “you make yourself sick just with your mind” (I1 P2, Male). Another participant reinforced this idea by describing depression as something one must simply push through, positioning it as a test of mental strength, “I think I’m not very weak in my mind…I have to take care of myself, I have to take care of my children, of my wife, of my parents. And then it [the depression] goes away” (I1 P6, Male). For these participants, depression was something they believed they should control, not just for themselves but for their families. For some, this mindset appeared to create resistance to depression care, as seeking treatment was described as an admission of personal failure. One participant described outright rejecting the PCP resources, saying they simply “refused [depression resources] a couple of times” (I1 P3, Male). Another participant hesitated to take medications, citing concerns about dependence, “He [my PCP] prescribed me some pills, but to be honest, I barely took them…Because I didn’t want to depend on a medication” (I1 P6, Male). Patients’ depression beliefs appeared to influence their willingness to accept external interventions. Instead of formal depression treatment, participants who viewed depression as a personal failing often favored self-directed strategies for symptom management. One participant described using dietary and complementary health strategies rather than engaging with medical interventions, “I started my daily diet by drinking a cup of tea, eating a piece of orange, having boiled lemon water. And that’s how I got rid of the depression” (I1 P2, Male).

Other participants described depression as something beyond an individual’s control, often associating it with a hereditary or genetic source. One participant described her depression as deeply rooted in her family’s history, noting its multigenerational presence, “I think that sometimes it is something, something genetic, sometimes caused by the problems that, that you may have…I’ve always had like episodes of, of…So I don’t know, maybe mine is more genetic, I don’t know because I see that my mom, mom was never diagnosed with anything, but she is very depressive…My son is like that too” (I1 P9, Female). Another participant reinforced this idea by framing depression as inevitable, or as something out of their control, “you can’t control mental things, you can’t control how things lead to depression for some reason or another” (I1 P4, Female). For these participants, depression is perceived as having strong biological roots. This belief was associated with a stronger willingness to engage in depression care, as seeking and accepting treatment did not carry implications about personal strength or willpower. One participant expressed hesitation about taking medication but was highly motivated to engage in psychotherapy, “I don’t want to see myself in that situation, that’s why I look for help, even if I have to knock on one door and another and another until I find where they can help me every time I want to talk about my issues” (I1 P4, Female). The belief that depression is rooted in biology or genetics appeared to reduce stigma and encourage help-seeking, particularly for psychotherapy, among our participants.

## Discussion

Our study explored how Latine primary care patients conceptualize depression and engage with depression care. Findings highlight how trust in PCPs, coping strategies rooted in cultural practices and PCP validation of these strategies, and language dynamics shape patients’ mental health experiences. Trust in PCPs was influenced by perceptions of comprehensive care, language and gender concordance, and the quality of the patient-provider relationship. Participants described diverse coping strategies, often prioritizing leisure, family support, and herbal remedies before seeking professional treatment but also using those in tandem with medications or therapy. Additionally, there was wide variation in patients’ recollection and understanding of their depression diagnosis and of the etiology of depression. Some viewed depression as genetic or external to their control, while others expressed a belief that conceptualized depression as a reflection of their willpower and self-discipline. These varied conceptualizations appeared to influence patient willingness to seek and remain engaged in treatment.

Our findings deepen existing research on stigma, trust, and culturally responsive care by illustrating how these dynamics play out in primary care settings serving Latine populations.[15][16][17] Participants emphasized the value of PCP behaviors that signaled attentiveness, personal connection, and cultural sensitivity, qualities that fostered trust and opened the door to conversations about mental health.[11] As prior studies have noted, trust is foundational to care engagement, especially when mental health is addressed outside of specialty settings.[23] Our study adds nuance by showing that trust is shaped not only by physician demeanor and communication style, but also by the validation of patients’ preferred coping strategies. While all the patients had a documented diagnosis of depression, some patients reported not having a diagnosis or did not remember these discussions. This is consistent with the literature that discussions about mental health can be more subtle,[24] leaving patients with lingering questions or doubts.

Implications for care improvement include the need to actively normalize mental health discussions in primary care settings. Participants often did not raise mental health concerns unless trust had been clearly established, underscoring the value of brief, rapport-building exchanges and culturally responsive communication.[25]. Ultimately, ensuring patients have a clear understanding of their depression diagnosis is essential,[8] highlighting the need for effective patient education and diagnostic communication strategies that are feasible within the time constraints of primary care. Furthermore, addressing structural barriers in critical. System-level investments in bilingual staff, and cultural humility training may address persistent barriers in language access [13] and improve engagement in depression care. Cultural humility promotes lifelong learning, self-reflection, and recognition of power dynamics in care.[26] It encourages PCPs to approach patients as experts in their own values and experiences, fostering trust and improving communication in diverse settings. Other participants stressed the importance of clinicians recognizing and validating culturally congruent coping strategies. Integrating these practices through shared decision-making may foster engagement and adherence to care plans that incorporate both biomedical and culturally-rooted approaches.

Our study has limitations. Our sample size was small; however, we reached thematic saturation with our sample and our sample size is consistent with prior qualitative research studies on similar topics. Our participants were already engaged in primary care which may not reflect the experiences of those excluded or disengaged from the healthcare system. The generalizability of patient coping strategies may be limited to patients with milder symptoms, but point to the importance of early diagnosis when these strategies could be most helpful. However, mean PHQ-9 scores among our participants indicated ‘moderate depression.’ Findings may not be generalizable to all Latine subgroups, as cultural beliefs and care-seeking behaviors vary across country of origin, and acculturation. Future research should seek to explore how levels of acculturation shape depression conceptualization and treatment preferences, as well as assess interventions that enhance trust and culturally competent communication, particularly about depression diagnosis, in primary care.

## Conclusions

This study identified key barriers and facilitators influencing Latine patient engagement in mental health care. These include the role of trust and relationship-building with PCPs, culturally congruent coping strategies, and variability in understanding and accepting depression diagnoses. Addressing these barriers through linguistically and culturally responsive approaches may improve mental health outcomes among Latine patients.

## Supplementary Material

Supplementary Files

This is a list of supplementary files associated with this preprint. Click to download.

- Appendix1InterviewGuide.docx
- Appendix2TargetedCodebook.docx
- Appendix3InformedConsent.docx
- Appendix4AbbreviationsList.docx
- LatinxStudyCOREQChecklist.docx

## Data Availability

The datasets generated and/or analyzed during the current study are not publicly available because individual consents did not include sharing of transcripts, but are available from the corresponding author on reasonable request.
